# CGF Membrane Promotes Periodontal Tissue Regeneration Mediated by hUCMSCs through Upregulating TAZ and Osteogenic Differentiation Genes

**DOI:** 10.1155/2021/6644366

**Published:** 2021-08-04

**Authors:** Wenjing Li, Feifei Wang, Fusheng Dong, Zhiyong Zhang, Peng Song, Huizhen Chen, Jie Wang

**Affiliations:** ^1^Department of Oral Pathology, School and Hospital of Stomatology, Hebei Medical University, The Key Laboratory of Stomatology, 419 East Zhongshan Road, Shijiazhuang Hebei 050017, China; ^2^Department of Oral Medicine, The Second Hospital of Hebei Medical University, China; ^3^Department of Stomatology, The Third Hospital of Hebei Medical University, China; ^4^Department of Oral and Maxillofacial Surgery, School and Hospital of Stomatology, Hebei Medical University, China

## Abstract

Concentrated growth factor (CGF) membranes are widely used in basic and clinical research of soft and hard tissue regeneration, but its effect on periodontal tissue regeneration is less studied. This study explored the role of CGF membranes in periodontal tissue regeneration mediated by human umbilical cord mesenchymal stem cells (hUCMSCs). HUCMSCs and human periodontal ligament fibroblasts (HPLFs) were extracted and identified by microscope and flow cytometry. The effects of the extracted CGF membrane on cell viability, osteogenic differentiation ability, osteopontin (OPN) expression, alkaline phosphatase (ALP) content, and osteogenic differentiation-related genes (Runt-related transcription factor 2 (RUNX2); osteocalcin (OCN); ALP), Tafazzin (TAZ) expression, and nuclear transfer were examined by MTT assay, alizarin red staining, immunofluorescence, enzyme-linked immunosorbent assay (ELISA), quantitative real-time polymerase chain reaction (qRT-PCR), and Western blot. Rescue experiments were performed to examine the effects of TAZ transfection and cell coculture. In the identified hUCMSCs (positive expressions of CD29, CD44, CD146, and CD105), overexpressed TAZ (pc-TAZ) enhanced the promotive effect of CGF membrane on cell viability, cell cycle, mineralization, ALP content and expressions of OPN, TAZ and osteogenic differentiation-related genes, and nuclear transfer. However, silencing TAZ showed opposite effects. The coculture of hUCMSCs and HPLFs further promoted the basic biological functions of HPLFs by upregulating osteogenic differentiation-related genes and COL-1 but downregulated MMP1 expression. Pc-TAZ could enhance the effect of CGF membrane on promoting periodontal tissue regeneration. CGF membrane promoted periodontal tissue regeneration through upregulating TAZ and osteogenic differentiation-related genes.

## 1. Introduction

Teeth are the hardest organs in the human body, but periodontal tissues are vulnerable to external stimuli under the influence of various external factors. Lesions in the periodontal tissues could cause periodontitis, leading to the destruction and absorption of periodontal ligaments, cementum, alveolar bone, and other dental support tissues [1, 2]. At present, the main periodontal treatment in clinical practice is to remove tartar, plaque, and diseased root surface to eliminate local inflammation of periodontal tissues and prevent further progression [3]. Currently, tissue regeneration technology has become a promising strategy for the treatment of periodontal tissue lesions [4]. Since the guided tissue regeneration (GTR) technology was proposed in 1980, tissue regeneration engineering has attracted much research attention in the past ten years [5, 6]. Mesenchymal stem cells play an important role in maintaining tissue self-renewal and self-repair and, therefore, are often used as seed cells for tissue regeneration engineering research [7].

Human umbilical cord mesenchymal stem cells (hUCMSCs) are a type of adult stem cells in the mesenchymal stem cell family, with a high multidirectional differentiation potential and self-renewal ability [8]. Studies confirmed that hUCMSCs could be successfully induced to differentiate into osteoblasts, adipocytes, nerve cells, skin fibroblasts, and other tissue cells *in vitro* [8, 9]. HUCMSCs can express common stem cell surface markers such as CD29, CD44, and CD105 and also markers of embryonic stem cells such as NANOG and DNMT3B [10]. These features of hUCMSCs allow it to become seed cells widely studied in tissue regeneration engineering. Xue et al. [11] used liver homogenate supernatant to simulate the liver tissue microenvironment *in vivo* and finally induced hUCMSC to differentiate into hepatocytes; Yin et al. [12] found that infusion of hUCMSC caused localized *M*1 macrophages in the pancreas of type 2 diabetic mice to an anti-inflammatory *M*2-like state. However, there are few reports studying hUCMSC in the intervention of periodontal disease [13].

Concentrated growth factor (CGF) fibrin membrane is the third generation platelet enrichment [14]. Compared with previous generations of platelet enrichment, the preparation of CGF membranes does not require additional reagents to generate platelet activation or fibrin polymerization and is less likely to develop immune responses or cross infections [15, 16]. CGF membrane has been extensively studied in bone tissue engineering, soft tissue injury, cardiovascular disease, and nervous system diseases [17, 18] and used in periodontal surgery, extraoral surgery, and implants [19]. However, the roles of CGF fibrin membrane and hUCMSCs in periodontal tissue regeneration have not been reported.

TAZ, also known as the WWTR1 gene, is located on the human chromosome 3q23-q24 [20]. TAZ was originally discovered as a binding protein that can enter the nucleus and bind to transcription factors to activate transcription factor activity and promote gene expression [21]. TAZ functions in the conditional knockout of ephrinB1 in osteogenic progenitors to delay the process of endochondral ossification during fracture repair [22]. Whether the role of CGF fibrin membrane and hUCMSCs in periodontal tissue regeneration was related to TAZ was investigated in our research.

In this study, by preparing CGF fibrin membrane and extracting hUCMSCs and human periodontal ligament fibroblasts (HPLFs), the role and specific mechanisms of CGF fibrin membrane in periodontal tissue regeneration mediated by hUCMSCs were clarified.

## 2. Materials and Methods

### 2.1. Ethics Statement

All the umbilical cord tissues and the venous blood in this experiment were collected from the Department of Oral Medicine of The Second Hospital of Hebei Medical University and conducted under the approval from the Hospital Ethics Committee (No. 201810021FCK). The umbilical cords (*n* = 3) were obtained from cesarean women without any systemic diseases and used for the primary culture of hUCMSCs. HPLFs were extracted from the premolars (*n* = 6) obtained from of the periodontal healthy, caries-free young volunteers aged 12-28 years old, together with the venous blood. This study was approved by the Ethics Committee of The Second Hospital of Hebei Medical University (No. 201811013KQK). The sample provider and their guardian signed informed consent for the clinical study.

### 2.2. Cell Extraction and Culture

The excised umbilical cord tissues were rinsed in normal saline and placed in a DMEM culture medium (C11995500BT, Invitrogen, USA) containing 4% of penicillin and streptomycin (SV30010, Hyclone, USA) at 4°C. After the umbilical cord was fixed, the envelope tissues were separated from the blood vessels of the umbilical cord using a pair of ophthalmic scissors, and the obtained umbilical cord tissues were cut to 1 mm^2^. Type I collagenase (1 mL, C0130, Gibco, USA) and Dispase (1 mL, 17105041, Invitrogen, USA) were used to fully digest the tissues at 37°C. After 16 hours (h), human umbilical cord mesenchymal stem cells were added to a DMEM medium with 20% fetal bovine serum (SH30096.03, Hyclone, USA) to terminate the digestion and resuspend the cells. The finally harvested human umbilical cord mesenchymal stem cells were cultured in a Herocell C1 carbon dioxide incubator (RADOBIO, China) at 37°C with 5% CO_2_. HPLFs were taken from premolars provided by volunteers. Before tooth extraction, volunteers gargled 3% hydrogen peroxide solution (M164, Beijing Land Bridge Technology Co., Ltd., China) for 3 minutes (min), and then the surface of the crown was cleaned with 75% alcohol. The extracted teeth were immediately placed in a prechilled DMEM medium (C11995500BT, Invitrogen, USA) containing penicillin and streptomycin (SV30010, Hyclone, USA) at 4°C. The periodontal ligament tissues were scraped by a sterile blade, cut into 1 mm^3^ pieces and then transferred to a DMEM medium containing 20% FBS and cultured in a Herocell C1 carbon dioxide incubator (RADOBIO, China) at 37°C with 5% CO_2_. After the separation and culture of the two cells, the growth and morphology of the cells were observed under a light microscope (Leica DM4 B & DM6 B., Leica, Germany).

### 2.3. Identification of hUCMSCs

To confirm that the cells extracted were hUCMSCs, cell surface markers were analyzed using a CytoFLEX S flow cytometer (Beckman Coulter, USA). The cell density of hUCMSCs in the third logarithmic growth phase was adjusted to 1 × 10^6^ cells/mL and then aliquoted into EP tubes (100 *μ*L). One tube was added with rabbit anti-mouse IgG-PE as a negative control, and the remaining cell suspensions were added with Mesenchymal Stromal Cell Marker (CD44, CD45, CD90 CD29, CD105), Antibody Panel (ab93758, Abcam, UK), Anti-CD34 antibody (ab81289, Abcam, UK), and Anti-CD146 antibody (ab75769, Abcam, UK) and incubated at room temperature in the dark for 60 min. The antibody-incubated cells were resuspended in 500 *μ*L of PBS and then transferred to a flow cytometer to detect the levels of CD29, CD44, CD45, CD34, CD146, and CD105 on the cell surface.

### 2.4. Preparation of Concentrated Growth Factors (CGF) and Cell Treatment

The vacuum blood collection tube containing the venous blood of the healthy volunteer was prepared for differential centrifugation as follows for a total of 13 min at 4°C: acceleration for 30 seconds (s); at 2700 rpm for 2 min; at 2400 rpm for 4 min; at 2700 rpm for 4 min; at 3000 rpm for 3 min; deceleration for 36 s and terminated. The blood in the blood collection tube was divided into three layers, the middle of which was the CGF gel layer. By discarding the upper serum and separating the lower red blood cells, the CGF fibrin membrane was removed, cut into 3 × 3 mm^2^, and placed in sterile physiological saline for later use. HUCMSCs were divided into the control group (without CGF fibrin membrane) and CGF1 group (with a piece of CGF fibrin membrane). The prepared CGF was used for cocultivation with hUCMSCs (1 × 10^4^ cell/well). When the degree of cell fusion reached 80%, the hUCMSCs were used for subsequent studies.

### 2.5. Construction and Transfection of Lentiviral Plasmids

In order to prepare lentiviral plasmids for overexpressed Tafazzin (pc-TAZ) and knockdown TAZ (shTAZ), we first amplified the overexpressed TAZ and shTAZ (5′-GCCATGTGATTACTTTTCCATCA-3′), and the amplified double-stranded structures were ligated into overexpression plasmid vectors (pcDNA3.1 vector, HG-VPI0001, Invitrogen, USA) and shRNA plasmid vectors (pGpU6/GFP/Neo vector, CO2007, GenePharma, China). 10 *μ*L of the recombinant plasmid and 800 *μ*L of LB medium (L1015, Solarbio, China) were added to competent bacteria and verified by sequencing of plasmid positive clones. Cell transfection was conducted by incubating virus supernatants containing recombinant plasmids collected with 293T cell-packed lentivirus preparation methods with the corresponding cells to construct stably overexpressed TAZ or shTAZ cell lines.

### 2.6. MTT Assay

The cells were carefully digested by 0.25% trypsin-EDTA solution (25200-056, GIBCO, USA) for 24 h. A cell suspension (100 *μ*L) adjusted to a cell density of 5 × 10^3^ cells/mL was transferred to a 96-well plate. 90 *μ*L of cell culture fluid and 10 *μ*L of MTT solution were added and incubated for 4 h, and then 100 *μ*L of dimethyl sulfoxide (DMSO) was added to the cells. After 24, 48, and 72 h, the cells were examined for absorbance at 490 nm using a Molecular Devices SpectraMax iD5 multifunctional microplate reader (USA).

### 2.7. Alizarin Red Mineralization Experiment

The cells (2 × 10^4^ cell/mL) were seeded into 100 mm dish, and the culture medium was replaced by osteogenic induction fluid (GC1009, Servicebio, China). After 21 days of incubation at 37°C and 5% CO_2_, the cells were washed 3 times with PBS and fixed with 4% paraformaldehyde for 20 min. Alizarin Red S staining solution (G1452-100mL, Solarbio, China) was used to stain the cells at room temperature for 30 min. The washed cells were observed for the staining with an optical microscope (Leica DM4 B & DM6 B., Leica, Germany). Alizarin Red was extracted by destaining with hexadecyl pyridinium chloride monohydrate. Mineral accumulation was quantified on a Molecular Devices SpectraMax iD5 multifunctional microplate reader at 562 nm. The data were normalized by the total protein concentration detected in a duplicate plate.

### 2.8. Immunofluorescence Staining

Immunofluorescence staining was used to determine HPLFs and osteopontin (OPN) expression. The cells were first washed with PBS and fixed with 4% paraformaldehyde for 20 min. The cells permeabilized with 0.1% TritonX-100 (T8787-50ML, Sigma-Aldrich, USA) were blocked by 5% goat serum for 30 min. Anti-Vimentin antibody (1 : 500, ab137321, Abcam, UK), Anti-Cytokeratin antibody (1 : 500, ab756, Abcam, UK), and Anti-OPN antibody (1 : 1000, ab8448, Abcam, UK) were separately added to the cells and incubated overnight at 4°C. Goat Anti-Rabbit IgG H&L (Alexa Fluor® 488) (1 : 5000, ab150077, Abcam, UK) and Goat Anti-Mouse IgG H&L (Alexa Fluor® 488) preadsorbed (1 : 5000, ab150117, Abcam, UK) were combined with primary antibody for color rendering for 2 h at room temperature. Finally, the nuclei were stained with 4′, 6-diamidino-2-phenylindole (DAPI). The mounted sections were examined by Leica DM4 B & DM6 B Upright fluorescence biological microscope (Leica, Germany) to observe the fluorescence expression.

### 2.9. Detection of Alkaline Phosphatase (ALP)

The content of ALP was detected by an enzyme-linked immunosorbent assay (ELISA) method. Cells with a density of 1 × 10^5^ cells/mL were seeded into 12-well plates at 37°C for 24 h. The content of ALP in the cells was measured using an ALP kit (EH2618; Fine Biotech, China) at day 0 and 21.

### 2.10. Extraction of Total RNA and Quantitative Real-Time Polymerase Chain Reaction (qRT-PCR)

By referring to a previous research [23], total RNA was extracted by applying the TRIzol method. Briefly, TRIzol (1 mL, 15596018, Invitrogen, USA) was used to separate total cellular RNA from other components. RNA was formed to a precipitate using Trichloromethane (200 *μ*L, T819285, MACKLIN, China), isocyanate propanol (500 *μ*L, I811932, MACKLIN, China), and 75% ethanol (E809064, MACKLIN, China). RNA concentration was measured by NanoDrop 2000 (ND-LITE-PR; Thermo Fisher, USA). RNA was then reverse-transcribed into cDNA by a PrimeScript RT reagent Kit (RR047A, TaKaRa, Japan). QRT-PCR was conducted in the QuantStudio 3 real-time PCR system (Thermo Fisher, USA) under the following conditions: predenaturation at 95°C for 10 min, denaturation at 95°C for 15 s, and annealing at 60°C for 1 min, for a total of 40 cycles. The primer sequences used in this experiment were as follows: runt-related transcription factor 2 (RUNX2)-F, 5′-TCTCTGGTTTTTAAATGGTTA-3′, and RUNX2-R, 5′-CTTGTACCCTCTGTTGTAAAT-3′; osteocalcin (OCN)-F, 5′-ACCGAGACACCATGAGAG-3′, and OCN-R, 5′-CTGGGTCTCTTCACTACCT-3′; ALP-F, 5′-AGACCTTCATAGCGCACGTC-3′, and ALP-R, 5′-ACCTTTGGCTCTCGACCAG-3′; *β*-actin (internal reference)-F, 5′-ATTGGCAATGAGCGGTTC-3′, and *β*-actin-R, 5′-GGATGCCACAGGACTCCA-3′. mRNA expressions of genes were quantified through 2^-*ΔΔ*CT^.

### 2.11. Western Blot

By referring to previous research [24], RIPA Lysis Buffer (C05-01001, Bioss, China) was used for total cell protein extraction. In order to detect the distribution of genes in the nucleus and cytoplasm, Cytoplasmic and Nuclear Fractionation kit (SC-003, INVENT, USA) was used to isolate the cytoplasm and nucleus and further extract total proteins. The protein was transferred to the nitrocellulose filter membrane by the method of sodium dodecyl sulfate–polyacrylamide gel electrophoresis (SDS–PAGE, 10%). The process of protein blocking and antibody incubation was completed briefly as follows: the protein on the NC membrane was blocked by the blocking solution for 2 h. The primary antibodies of related genes (TAZ, 1 : 500, ab224239, 44 kDa, Abcam, UK; collagen-1, COL-1, 1 : 500, ab64883, 181 kDa, Abcam, UK; matrix metalloprotein 1, MMP1, 1 : 1000, ab137332, 54 kDa, Abcam, UK; *β*-actin, 1 : 5000, ab8226, 42 kDa, Abcam, UK; Lamin B1, 1 : 1000, ab220797, 70 kDa, Abcam, UK) were bound to the corresponding antigen in the protein overnight at 4°C. Corresponding secondary antibodies (Goat anti-rabbit IgG, 1 : 5000, ab205718, Abcam, UK; Goat anti-mouse IgG, 1 : 5000, ab205719, Abcam, UK) were bound to the primary antibody for 1.5 h. ECL luminescent fluid (WBK1S0100, Millipore, USA) and gel imaging system (GelDoc XR+, Bio-Rad, USA) were used for the final detection of protein expressions.

### 2.12. Cell Coculture

In order to explore the role of human umbilical cord mesenchymal stem cells in the growth of HPLFs, the human umbilical cord mesenchymal stem cells in the upper chamber and the HPLFs in the lower chamber were cocultured using a Transwell chamber (8 *μ*m, BD Biosciences, USA). The HPLFs of the upper chamber were grouped and treated accordingly as follows: blank (no treatment), CGF (2 pieces of CGF), vector (human umbilical cord mesenchymal stem cells transfected with empty vector), CGF + vector (2 slices of CGF and human umbilical cord mesenchymal stem cells transfected with an empty vector), shTAZ (human umbilical cord mesenchymal stem cells transfected with shTAZ), CGF + shTAZ (2 slices of CGF and human umbilical cord transfected with shTAZ mesenchymal stem cells), pc-TAZ (human umbilical cord mesenchymal stem cells transfected with pc-TAZ), and CGF + pc-TAZ (2 slices of CGF and human umbilical cord mesenchymal stem cells transfected with pc-TAZ). The cells were cocultured in a cell incubator at 37°C with 5% CO_2_ for 48 h.

### 2.13. Cell Migration Assay

The back of the 6-well plate was marked with a plurality of parallel lines spaced 1 cm apart between two lines. Cell suspensions from different groups after trypsinization were added to 6-well plates and incubated overnight at 37°C. Next, a pipette was used to vertically mark the lines, and the cells were removed. Subsequently, the cells were incubated with serum-free DMEM medium for 24 h, 48 h, and 72 h. Cell migration was recorded by microscope observation.

### 2.14. The Cell Cycle

Cell cycle detection was performed using a cell cycle detection kit (KGA511, Keygen, China). According to the instructions, the cells immobilized in 70% ethanol overnight were mixed with the RNase reagent at 37°C for 1 h. The PI reagent was then used to stain the cells for 30 min. Finally, the mixed solution was analyzed by CytoFLEX S flow cytometer (Beckman Coulter, USA).

### 2.15. Data Analysis

Experiments were performed in independent triplicate. Statistical analysis was performed with SPSS 19.0 (Chicago, IL, USA). Statistical significances between groups were determined with one-way ANOVA and Tukey's *t* test. *p* < 0.05 signified significant statistics.

## 3. Results

### 3.1. The Identification of hUCMSCs

**Figure 1:**
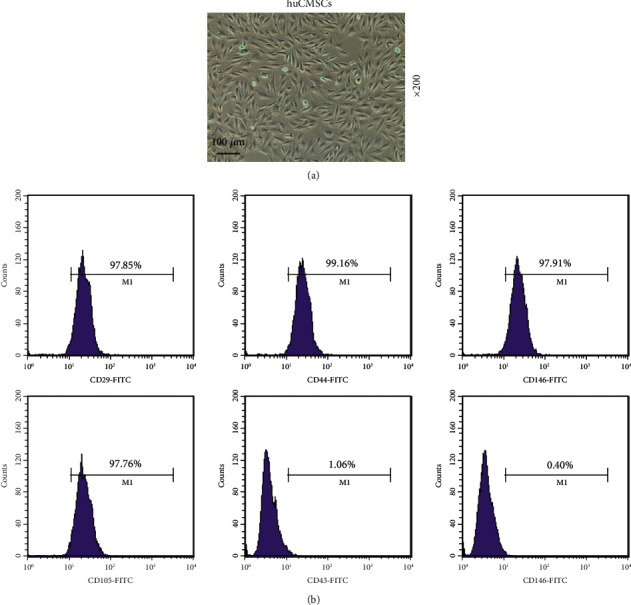
Identification of human umbilical cord mesenchymal stem cells (hUCMSCs). (a) Cell morphology was observed under a microscope, under 200 × magnification. (b) Flow cytometry was used to identify cell surface markers. All experiments were performed in independent triplicate.

The extracted hUCMSCs were identified. Observation with a microscope showed that the extracted cells were long spindle-shaped and polarized (Figure 1(a)). The results of flow cytometry analysis (Figure 1(b)) demonstrated that the expression rates of stem cell surface markers CD29, CD44, CD146, and CD105 in the extracted cells were 97.85%, 99.16%, 97.91%, and 97.76%, respectively, and the hematopoietic marker CD34 (1.06%) and CD45 (0.40%) were negative. The above experimental results fully proved the cells extracted were hUCMSCs.

### 3.2. CGF Fibrin Membrane Promoted the Viability, Osteogenic Differentiation, OPN Expression, and ALP Content of hUCMSCs

**Figure 2:**
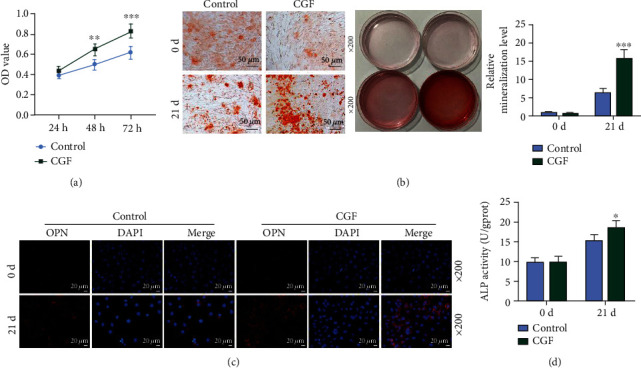
Concentrated growth factors (CGF) fibrin membrane promoted the viability, osteogenic differentiation, osteopontin (OPN) expression, and ALP content of hUCMSCs. (a) Cell viability was measured by MTT assay. (b) Alizarin staining was used to identify the differentiation of stem cells into osteoblasts, under 200 × magnification. Mineral accumulation was quantified. (c) Immunofluorescence staining was used to detect OPN expression, under 200 × magnification. (d) The content detection of alkaline phosphatase (ALP). All experiments were performed in independent triplicate. ^∗^*p* < 0.05, ^∗∗^*p* < 0.01, ^∗∗∗^*p* < 0.001 vs. control.

By employing MTT assay, alizarin staining, immunofluorescence staining, and ELISA assay, we investigated the effect of CGF fibrin membranes on hUCMSCs. CGF fibrin membrane was found to significantly promote cell viability (Figure 2(a), *p* < 0.01). As shown in Figure 2(b), after the intervention of CGF fibrin membrane, the red mineralized nodules formed in hUCMSCs on day 21 were obviously more than those in the control group (*p* < 0.001). The detection results of OPN were shown in Figure 2(c), on day 21, it could be observed that the positive expression of red OPN in the visual field of the CGF group was significantly higher than that of the control group. CGF fibrin membrane also increased ALP content in the cells (Figure 2(d), *p* < 0.05).

### 3.3. CGF Fibrin Membrane Upregulated the Expression of Osteogenesis-Related Genes and TAZ and Promoted the Nuclear Transfer of TAZ

**Figure 3:**
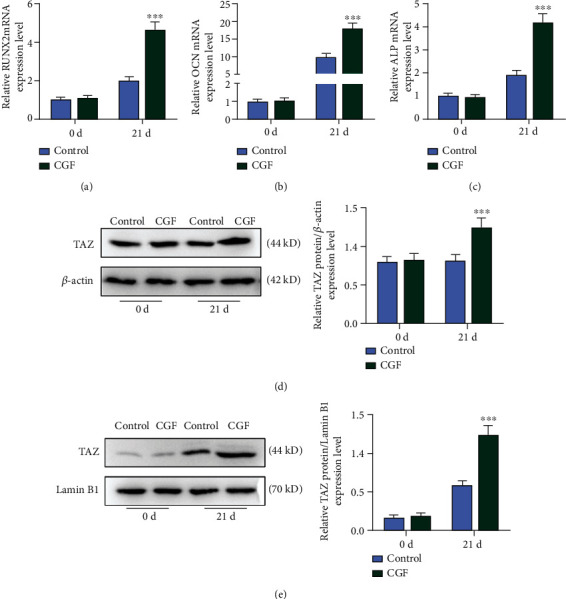
CGF fibrin membrane upregulated the expression of osteogenesis-related genes and transcriptional coactivator with PDZ-binding motif (TAZ) and promoted the nuclear transfer of TAZ. (a–c) The mRNA expressions of osteogenesis-related genes were analyzed by Quantitative Real-Time Polymerase Chain Reaction (qRT-PCR). *β*-Actin was employed as an internal control. (d and e) TAZ protein expression in cytoplasm and nucleus was detected by Western blot. *β*-Actin and Lamin B1 were employed as an internal control. All experiments were performed in independent triplicate. ^∗∗∗^*p* < 0.001 vs. control.

CGF fibrin membranes could also regulate the expressions of osteogenic-related genes in cells in addition to its role in affecting basic physiological functions of hUCMSCs. On the 21st day of cell culture, the mRNA expressions of RUNX2, OCN, and ALP were significantly upregulated in the CGF group compared with the control group (Figures 3(a)–3(c), *p* < 0.001). The detection of TAZ expression in the nucleus and cytoplasm showed that TAZ expression in the cytoplasm and nucleus was significantly upregulated after the intervention of CGF fibrin membrane (Figures 3(d) and 3(e), *p* < 0.001). These results indicated that CGF fibrin membrane promoted the nuclear transfer of TAZ.

### 3.4. The Effects of Overexpressed or Silencing TAZ on the Expressions of Genes Related to Osteoblast Differentiation, Osteogenesis, and TAZ

**Figure 4:**
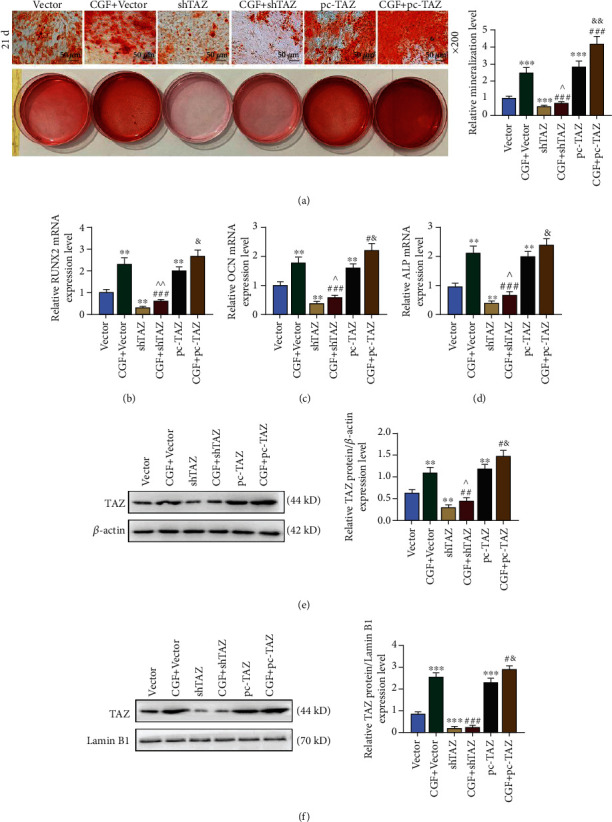
The effects of overexpressed or silenced TAZ on the expressions of osteoblast differentiation, osteogenesis-related genes, and TAZ. (a) Alizarin staining was used to identify the differentiation of stem cells into osteoblasts, under 200 × magnification. Mineral accumulation was quantified. (b–d) The mRNA expressions of osteoblast-related genes were analyzed by qRT-PCR. *β*-Actin was employed as an internal control. (e and f) TAZ protein expression in cytoplasm and nucleus was detected by Western blot. *β*-Actin and Lamin B1 were employed as internal controls. All experiments were performed in independent triplicate. ^∗∗^*p* < 0.01, ^∗∗∗^*p* < 0.001 vs. vector; ^#^*p* < 0.05, ^##^*p* < 0.01, ^###^*p* < 0.001 vs. CGF + vector; ^^^*p* < 0.05, ^^^^*p* < 0.01 vs. shTAZ; ^&^*p* < 0.05, ^&&^*p* < 0.01 vs. pc-TAZ.

The effects of overexpressed or silencing TAZ on hUCMSCs were investigated using constructed plasmids. The results of Alizarin staining (Figure 4(a)) showed that compared with the vector group, the red mineralized particles in the shTAZ group were decreased (*p* < 0.001), whereas the red mineralized area in the pc-TAZ group was increased (*p* < 0.001). CGF fibrin membrane intervention improved the condition of the shTAZ group (Figure 4(a), *p* < 0.001) and increased the mineralized area of the pc-TAZ group (Figure 4(a), *p* < 0.01). ShTAZ inhibited the mRNA expressions of RUNX2, OCN, and ALP compared with the vector group (Figures 4(b)–4(d), *p* < 0.01), whereas pc-TAZ showed the opposite regulatory effect (Figures 4(b)–4(d), *p* < 0.01). The CGF fibrin membrane also reversed the effect of shTAZ but promoted the effect of pc-TAZ (Figures 4(b)–4(d), *p* < 0.05). By detecting TAZ protein expression in cytoplasm and nucleus, we found that shTAZ inhibited TAZ protein expression in both cytoplasm and nucleus (Figures 4(e) and 4(f), *p* < 0.01), whereas pc-TAZ produced the opposite effect (Figures 4(e) and 4(f), *p* < 0.01). The intervention of CGF fibrin membrane was opposite to shTAZ and showed a promoting effect on pc-TAZ (Figures 4(e) and 4(f), *p* < 0.05).

### 3.5. The Identification of HPLFs

**Figure 5:**
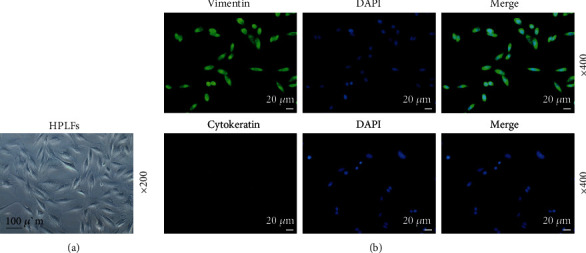
Identification of human periodontal ligament fibroblasts (HPLFs). (a) Cell morphology was observed under a microscope, under 200 × magnification. (b) Immunofluorescence staining was used to detect the expressions of Vimentin protein and Cytokeratin protein, under 400 × magnification. All experiments were performed in independent triplicate.

We identified the extracted human periodontal fibroblasts. Our observation with a microscope showed that the morphology of the extracted cells was mainly spindle-shaped with varied length of the cell protrusions, and the cell bodies were full, showing a typical fibroblastic morphology (Figure 5(a)). Immunofluorescence staining results demonstrated that Vimentin protein was positive, while Cytokeratin protein was negative (Figure 5(b)). The above experimental results fully proved that the cells extracted were human periodontal fibroblasts.

### 3.6. The Effects of CGF Fibrin Membrane, pc-TAZ, and shTAZ on the Nuclear Transfer of TAZ, the Cell Viability, and Migration

**Figure 6:**
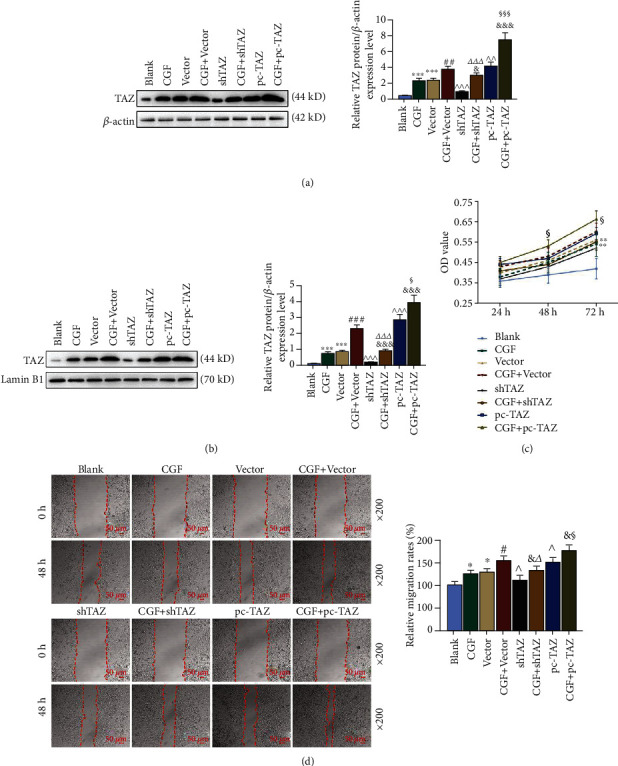
The effects of CGF fibrin membrane, pc-TAZ, and shTAZ on the nuclear transfer of TAZ, cell viability, and migration. (a and b) TAZ protein expression in cytoplasm and nucleus was detected by Western blot. *β*-Actin and Lamin B1 were employed as an internal control. (c) Cell viability was measured by MTT assay. (d) Cell migration was detected by Scratch assay, under 200 × magnification. All experiments were performed in independent triplicate. ^∗^*p* < 0.05, ^∗∗^*p* < 0.01, ^∗∗∗^*p* < 0.001 vs. blank; ^#^*p* < 0.05, ^##^*p* < 0.01, ^###^*p* < 0.001 vs. CGF; ^^^*p* < 0.05, ^^^^*p* < 0.01, ^^^^^*p* < 0.001 vs. vector; ^&^*p* < 0.05, ^&&&^*p* < 0.001 vs. CGF + vector; ^△^*p* < 0.05, ^△△△^*p* < 0.001 vs. shTAZ; ^§^*p* < 0.05, ^§§§^*p* < 0.001 vs. pc-TAZ.

**Figure 7:**
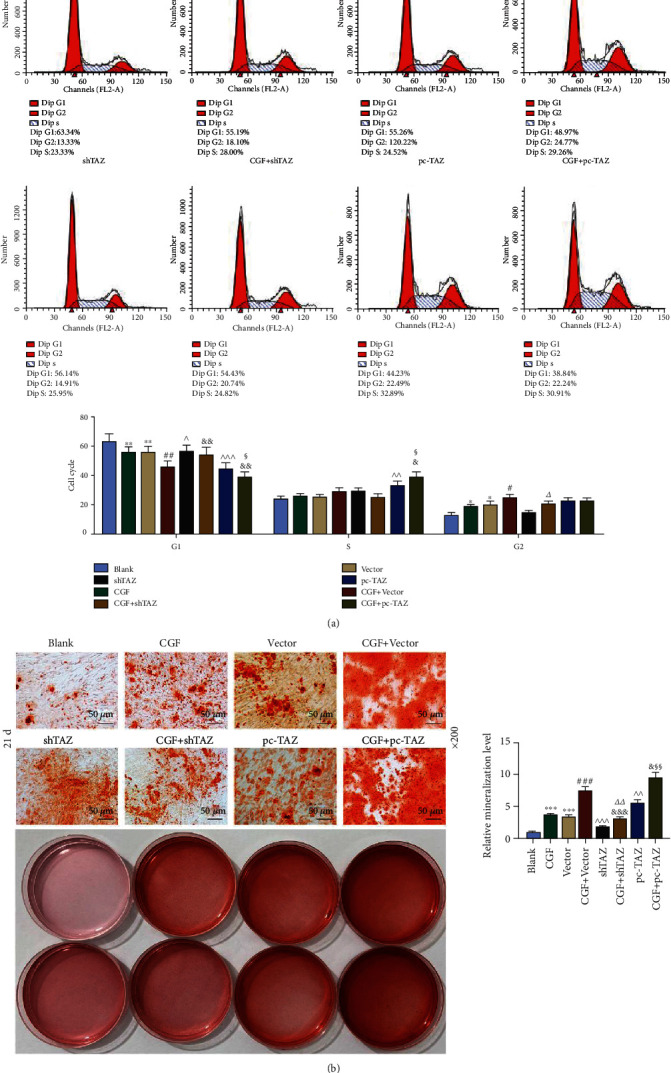
The effects of CGF fibrin membrane, pc-TAZ and shTAZ on cell cycle and differentiation of coculture. (a) Cell cycle changes were detected by flow cytometry. (b) Alizarin staining was used to identify cell mineralization, under 200 × magnification. Mineral accumulation was quantified. All experiments were performed in independent triplicate. ^∗^*p* < 0.05, ^∗∗^*p* < 0.01, ^∗∗∗^*p* < 0.001 vs. blank; ^#^*p* < 0.05, ^##^*p* < 0.01, ^###^*p* < 0.001 vs. CGF; ^^^*p* < 0.05, ^^^^*p* < 0.01, ^^^^^*p* < 0.001 vs. vector; ^&&^*p* < 0.01, ^&&&^*p* < 0.001 vs. CGF + vector; ^△^*p* < 0.05 vs. shTAZ; ^§^*p* < 0.05, ^§§^*p* < 0.01 vs. pc-TAZ.

The detection of TAZ expression in the nucleus and cytoplasm showed that TAZ expression in the cytoplasm and nucleus was significantly upregulated after the intervention of CGF fibrin membrane or hUCMSCs (Figures 6(a) and 6(b), *p* < 0.001). After coculture, hUCMSCs promoted the expression of TAZ (*p* < 0.01). The CGF fibrin membrane further enhanced the promotion by hUCMSCs and pc-TAZ (Figures 6(a) and 6(b), *p* < 0.05), but reduced the inhibition of shTAZ (Figures 7(a) and 7(b), *p* < 0.001). We cocultured hUCMSCs and HPLFs to study the effects of CGF fibrin membrane, pc-TAZ, and shTAZ on cell viability and migration. The experimental results showed that hUCMSCs promoted the viability and migration of HPLF cells (Figures 6(a) and 6(b), *p* < 0.05). CGF fibrin membrane increased the viability and migration of the cocultured cells (Figures 6(a) and 6(b), *p* < 0.05), noticeably, CGF fibrin membrane reversed the inhibitory effect of shTAZ and enhanced the promoting effect of PC-TAZ (Figures 6(a) and 6(b), *p* < 0.05).

### 3.7. The Effects of CGF Fibrin Membrane, pc-TAZ, and shTAZ on the Cell Cycle and Differentiation of the Cocultured Cells

We further analyzed cell cycle changes after coculture by performing flow cytometry. Compared with the blank group, each group showed different degrees of promoted cell cycle and reduced G1 phase arrest (Figure 7(a), *p* < 0.05). pc-TAZ also significantly promoted cell proliferation in S phase (Figure 7(a), *p* < 0.01), and such an effect was further enhanced by CGF fibrin membrane (Figure 7(a), *p* < 0.05). Cell mineralization results detected by alizarin were presented in Figure 7(b); after coculture, hUCMSCs promoted the mineralization of HPLFs (*p* < 0.001). The CGF fibrin membrane further enhanced the promotion by hUCMSCs and pc-TAZ (Figure 7(b), *p* < 0.01), but reduced the inhibition of shTAZ (Figure 7(b), *p* < 0.01).

### 3.8. The Effects of CGF Fibrin Membrane, pc-TAZ, and shTAZ on the Expressions of Cocultured Osteogenesis-Related Genes and Fibrosis-Related Genes

**Figure 8:**
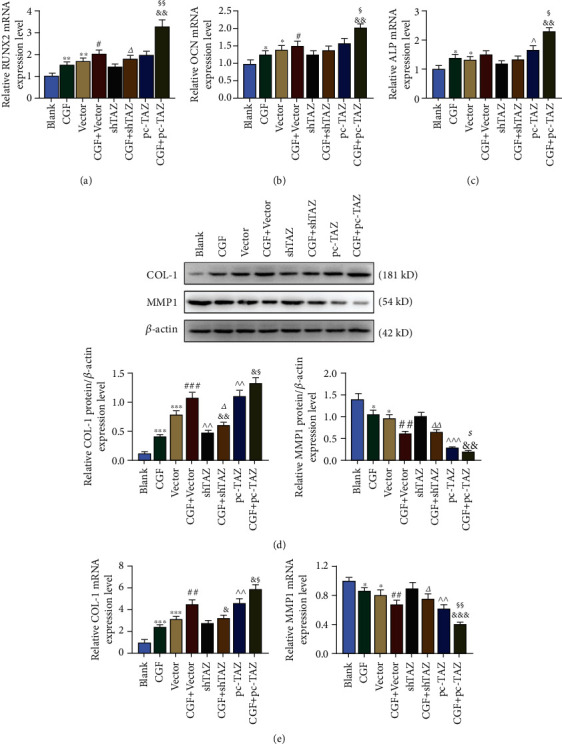
The effects of CGF fibrin membrane, pc-TAZ, and shTAZ on the expression of cocultured osteogenesis-related genes and fibrosis-related genes. (a–c) The mRNA expressions of osteogenesis-related genes were analyzed by qRT-PCR. *β*-Actin was employed as an internal control. (d) Protein expressions of fibrosis-related genes were detected by Western blot. *β*-Actin was employed as an internal control. (e) The mRNA expressions of fibrosis-related genes were analyzed by qRT-PCR. *β*-Actin was employed as an internal control. All experiments were performed in independent triplicate. ^∗^*p* < 0.05, ^∗∗^*p* < 0.01, ^∗∗∗^*p* < 0.001 vs. blank; ^#^*p* < 0.05, ^##^*p* < 0.01, ^###^*p* < 0.001 vs. CGF; ^^^*p* < 0.05, ^^^^*p* < 0.01, ^^^^^*p* < 0.001 vs. vector; ^&^*p* < 0.05, ^&&^*p* < 0.01, ^&&&^*p* < 0.001 vs. CGF + vector; ^△^*p* < 0.05, ^△△^*p* < 0.01 vs. shTAZ; ^§^*p* < 0.05, ^§§^*p* < 0.01 vs. pc-TAZ.

After cell coculture, compared with the blank group, the mRNA expressions of RUNX2, OCN, and ALP in the vector and CGF groups were all upregulated (Figures 8(a)–8(c), *p* < 0.05). Although pc-TAZ and shTAZ showed a promoting effect, the difference was not statistically significant. However, the intervention of CGF fibrin membrane significantly upregulated the expressions of osteogenic differentiation-related genes in the pc-TAZ group (Figures 8(a)–8(c), *p* < 0.05). The detection results of fibrosis-associated proteins COL-1 and MMP1 demonstrated that compared with the blank group, the expression of COL-1 was promoted to varying degrees in the CGF group, the vector group, the pc-TAZ group, and the shTAZ group (Figure 8(d), *p* < 0.01) However, the expression of MMP1 was inhibited (except in shTAZ group, Figure 8(d), *p* < 0.05), and CGF further enhanced the regulation of pc-TAZ (Figure 8(d), *p* < 0.05). Moreover, the mRNA expressions of the genes showed similar results (Figure 8(e)).

## 4. Discussion

Periodontitis was a common infective disease and a main cause of tooth loss. Local periodontal inflammation could destroy the integrity of the epithelium, causing periodontal pathogens to spread to the circulatory system [25]. At the same time, periodontal pockets contained a large amount of inflammatory mediators, especially those related to chronic inflammation, such as tumor necrosis factor *α*, interleukin, and prostaglandin, which could enter the blood circulation system from periodontal pockets and cause systemic inflammatory reactions [26, 27]. Therefore, timely and effective treatment was essential. Traditional periodontitis treatment focuses on inflammation control, aiming at preventing or delaying the disease progression, and it is still difficult to achieve a satisfactory regeneration of periodontal tissues [4]. The clinical effect of periodontal tissue regeneration treatment was greatly improved by the introduction of new technologies and materials such as guided tissue regeneration, bone transplantation, growth factors, and biological materials [3]. HUCMSCs derived from mesoderm are the seed cells of tissue regeneration engineering. Research showed that in a bone healing model based on cell regeneration, hUCMSCs show the same osteogenic characteristics as bone marrow mesenchymal stem cells [28] and could effectively promote fracture healing. In a study comparing the chondrogenic differentiation characteristics of hUCMSCs and bone marrow mesenchymal stem cells, the collagen production capacity of the former was three times higher than the latter [29]. Moreover, the previous study had successfully induced hUCMSCs *in vitro* to neural-like cells, liver-like cells, etc. [30, 31]. The important role of hUCMSCs in periodontal tissue regeneration has been gradually explored. Shang et al. [13] confirmed that hUCMSCs could promote the regeneration of periodontal tissues under inflammatory periodontitis conditions, similar to the role of human periodontal ligament stem cells. In this study, similarly, coculture of hUCMSCs and HPLFs promoted cell proliferation and differentiation, upregulated the expressions of RUNX2, OCN, ALP, and COL-1, and inhibited MMP1 expression.

RUNX2, OCN, and ALP are important regulatory genes of osteogenic differentiation. RUNX2 is a key regulatory factor regulating the differentiation of osteoblasts and osteoclasts to promote bone formation. By regulating the expressions of osteoblast-specific extracellular matrix protein genes and the osteoblast cycle, RUNX2 participates in the differentiation process of osteoblasts, promotes bone form, and inhibits bone resorption [32]. OCN, which is one of the most abundant noncollagenous bone proteins in the human body, is specifically expressed at the end of osteoblast differentiation and could regulate bone mineral deposition and metastasis promote osteoblast differentiation and maturation and bone cell formation [33, 34]. Mohamed-Ahmed et al. [35] found that bone marrow mesenchymal stem cells showed higher ALP activity and calcium deposition compared with adipose-derived stem cells. Combined with the findings from previous studies and experimental results of this study, the role of CGF in promoting osteogenic differentiation mediated by hUCMSCs is fully demonstrated.

In this study, CGF could promote cell viability, migration, and osteogenic differentiation; upregulate the expressions of RUNX2, OCN, ALP, and COL-1; and inhibit MMP1 expression, and such effects were enhanced with the intervention of pc-TAZ. Studies indicated that TAZ, which is widely distributed in tissues except thymus and peripheral lymphocytes, is high-expressed in a variety of tumor tissues and, therefore, is considered as a tumor-promoting gene [36]. Park et al. [37] demonstrated that downregulation of the Smad4/TAZ axis leads to delayed osteogenic differentiation of mesenchymal stem cells and increased adipogenesis. These findings further supported the reliability of this study.

CGF fibrin membrane is a blood agglutinate obtained by centrifuging venous blood specimens in a special centrifuge, which can produce fibrin with higher density and richer growth factors and is therefore widely used in many clinical treatments and tissue engineering [38]. Studies found that CGF promotes the proliferation and osteogenic differentiation of gingival mesenchymal stem cells, stimulates the bone differentiation of bone marrow stromal cells, and is an excellent biological material for bone regeneration [17, 39]. Our study not only confirmed that CGF fibrin membrane can promote hUCMSCs-mediated periodontal tissue regeneration but also revealed that the promotion is achieved by upregulating the expressions of TAZ and genes related to osteogenic differentiation. The current findings discovered that CGF fibrin membrane has the potential to serve as a bone regeneration biomaterial and provided new understandings to tissue regeneration treatment of periodontitis and other periodontal diseases.

## Data Availability

The analyzed data sets generated during the study are available from the corresponding author on reasonable request.
